# Implementation of Parenteral Nutrition Formulations with Increased Calcium and Phosphate Concentrations and Its Impact on Metabolic Bone Disease in Preterm Infants: A Retrospective Single-Centre Study

**DOI:** 10.3390/children12020172

**Published:** 2025-01-29

**Authors:** Sushma Sureshchandra, Rajesh Maheshwari, Tamara Nowland, James Elhindi, Lily Rundjan, Daphne D’Cruz, Melissa Luig, Dharmesh Shah, Gemma Lowe, Jane Baird, Pranav R. Jani

**Affiliations:** 1 Department of Neonatology, Westmead Hospital, Westmead, NSW 2145, Australia; sushma.sureshchandra@health.nsw.gov.au (S.S.); melissa.luig@health.nsw.gov.au (M.L.);; 2 Faculty of Medicine and Health, The University of Sydney, Sydney, NSW 2050, Australia; 3 Department of Radiology, The Children’s Hospital at Westmead, Westmead, NSW 2145, Australia; 4 Research and Education Network, Western Sydney Local Health District, Westmead, NSW 2145, Australia

**Keywords:** metabolic bone disease, MBDP, osteopenia of prematurity, parenteral nutrition

## Abstract

**Background:** Metabolic Bone Disease of Prematurity (MBDP) is common in extremely preterm infants (≤28 weeks gestation). Parenteral nutrition (PN) with higher calcium (Ca) and phosphorus (P) concentration started soon after birth may improve bone health in preterm infants. We compared the effect of two standard PN formulations on the incidence of MBDP and explored the predictive ability of biochemical markers for diagnosing MBDP. **Methods:** This retrospective study included eligible preterm infants ≤ 28 weeks gestation. Infants in group 1 (January 2016–December 2017) received PN 1 formulation with lower Ca (1.6 mmol/kg/day) and P concentration (1.4 mmol/kg/day). Infants in group 2 (June 2018–May 2020) received PN 2 formulation with higher Ca (2.3 mmol/kg/day) and P concentration (1.8 mmol/kg/day). We reviewed the biochemical and radiological investigations performed for diagnosing MBDP. **Results:** The incidence of MBDP reduced from 82.8% (77/93) in group 1 to 47.3% (27/57) in group 2. Grade 2–3 MBDP reduced significantly from 14% in group 1 to none in group 2 (*p* < 0.01). Serum phosphate < 1.5 mmol/L had a sensitivity of 79% and specificity of 77%, and alkaline phosphatase > 500 U/L showed a sensitivity of 72% and specificity of 71% for diagnosing radiological MBDP. There was no increase in hypercalcemia, hypophosphatemia or nephrocalcinosis from PN 2 formulation. **Conclusions:** A higher Ca and P concentration in PN reduced MBDP and eliminated grade 2–3 MBDP in our cohort without an increase in adverse events. Low serum phosphate and high serum alkaline phosphatase were the best predictors for diagnosing MBDP.

## 1. Introduction

Metabolic Bone Disease of Prematurity (MBDP) is a common and important morbidity in infants born extremely preterm. It is characterized by reduced bone mineralization due to inadequate calcium and phosphorus stores from reduced placental transfer of these minerals, inadequate intake, and high postnatal skeletal growth [1,2]. The true incidence of MBDP remains unknown due to a lack of consensus on the definition and screening practices for its diagnosis. Radiological changes linked to MBDP have been described in approximately 32% of very low birth weight infants (VLBW, <1500 g) and 54% of extremely low birth weight infants (ELBW, <1000 g) [3].

MBDP typically presents within 6–16 weeks after birth [4]. It may present subtly as poor extrauterine growth and increased ventilator dependency or, more dramatically, with bone fractures [5,6]. MBDP may have short and long-term consequences, such as dolichocephalic appearance of the head [7] and short stature in adulthood [8,9], although there is conflicting evidence on its association with the risk of osteoporosis in later life [10].

In 2013, the American Academy of Pediatrics (AAP) Committee on Nutrition published their guidelines for screening and management of MBDP. They suggested performing biochemical screening at 4–5 weeks after birth to identify MBDP in VLBW infants [11]. More recently, Grover et al. proposed a risk factor-based, tiered approach to screening for MBDP [12]. Despite this, considerable variation remains in screening, monitoring, and treating MBDP in VLBW infants [13,14]. Imaging is considered the gold standard test for diagnosing MBDP. However, a plain radiograph lacks the sensitivity to detect MBDP early as significant loss of bone mineralization is needed before characteristic changes are visible [15]. Access to other modalities such as Dual Energy X-ray Absorptiometry (DEXA) and ultrasound for diagnosing MBDP is limited. Therefore, there is potential utility of serum or urine biomarkers to predict MBDP early.

Parenteral nutrition (PN) is essential for providing nutrition to preterm infants before achieving full enteral feeding. Using higher early calcium (Ca) and phosphorus (P) in PN has prevented short-term bone strength decline in preterm infants [14]. To optimize bone formation in VLBW infants, PN supplementation should aim for higher Ca and P concentrations and an optimal Ca: P ratio to prevent precipitation. The AAP 2014 guideline recommended 1.5–2.0 mmol/kg/day of Ca and 1.5–1.9 mmol/kg/day of P intake, whereas the European Society for Paediatric Gastroenterology, Hepatology and Nutrition (ESPGHAN) 2005 guideline recommended 1.3–3 mmol/kg/day of Ca and 1.0–2.3 mmol/kg/day of P, with an optimal Ca:P ratio between 1.3–1.7 [13,16]. The current practice in all tertiary neonatal intensive care units (NICUs) in New South Wales and the Australian Capital Territory (ACT) is to use standard pre-mixed formulations based on the Australian Neonatal Parenteral Nutrition consensus group recommendations.

In our unit (XXXX NICU, which is a tertiary Neonatal Intensive Care Unit located in New South Wales, Australia), the first consensus formulations for standardized parenteral nutrition (PN 1) were used between July 2011 and March 2018, and the use of revised PN formulation (PN 2) was commenced in April 2018 based on the 2017 consensus group recommendations [17], which align with the ESPGHAN 2005 guidelines. The new PN formulation had a higher amino-acid, Ca, and P concentration with the change from inorganic to organic phosphate to allow for the increase in Ca and P content [17]. The major difference between the two PN formulations was the Ca:P ratio. PN 1 formulation, when administered at 135 mL/kg/day, provided 1.6 mmol/kg/day of Ca and 1.4 mmol/kg/day of P with a Ca:P ratio of 1.1 [18], while PN 2 formulation at the same rate would provide 2.3 mmol/kg/day of Ca and 1.8 mmol/kg/day of P with a Ca:P ratio of 1.3:1 [17]. There have been no studies to date evaluating the impact of these PN formulations on the bone health biomarkers in the preterm population.

### Objectives

The primary objective of this study was to compare the incidence of MBDP when using PN solutions with differing concentrations of calcium and phosphate. The secondary objective was to identify the sensitivity of available tests for diagnosing MBDP.

## 2. Materials and Methods

The study was conducted in the NICU at Westmead Hospital. In this retrospective study, we compared two groups of infants: Group 1 from 1 January 2016 to 31 December 2017 received PN 1 and Group 2 from 1 June 2018 to 31 May 2020 received PN 2 formulation. We included all infants born before 29 weeks’ gestation and who were admitted to our NICU within 24 h of birth. We excluded infants who were admitted beyond 24 h of age (as PN solution is not administered for outborn infants during transport), infants who were discharged or transferred to other hospitals, or who died before they could be investigated for MBDP (at 6 weeks of age). Eligible infants were identified from a prospectively maintained and verified Neonatal Intensive Care Units’ (NICUS) database. This database collates the maternal, perinatal, and neonatal clinical data of newborn infants admitted to all tertiary NICUs in New South Wales and the Australian Capital Territory. The data are collected and verified by designated audit officers following standardized definitions for clinical outcomes. Infants born between 1 January 2018 and 31 May 2018 (wash-out period) were excluded as they may have received both types of PN formulation. Additional data on laboratory biomarkers and plain radiographs were obtained from electronic medical records.

Our NICU’s practice is to screen infants born <29 weeks’ gestation for MBDP at 6 weeks after birth or at least two weeks after fortifying enteral feeds with human milk fortifier at 24 calories per 30 mL of breast milk. The screening for MBDP involves a plain radiograph of the long bone at the wrist or at the knee joint and biochemical tests: serum calcium, serum phosphate, alkaline phosphatase (ALP), 25-hydroxy vitamin D, parathyroid hormone (PTH) and tubular reabsorption of phosphate (TRP).

Human milk feeds were fortified with a bovine milk protein-based fortifier once the feeds were tolerated at a volume of 150 mL/kg/day. An important change that occurred during the second epoch was the introduction of pasteurized donor human milk (PDHM) in 2018 which was preferentially used in the second cohort where the mother’s own milk was not available. There were no other changes to the feeding protocol.

In this study, data was collected from 2 different timelines: Ca, P and ALP in the first 2 weeks of life whilst the infants were receiving PN (each of which is categorised as low, normal or high) and MBDP screening investigations (Ca, P, ALP, PTH, Vitamin D, TRP, X-rays of the wrist or knee) at 6 weeks of age or at 2 weeks after full fortification. A comparison of biochemical variables such as serum calcium and phosphate levels was performed during the administration of both PN formulations. The values were characterised as low, normal or high, based on the local laboratory-provided reference values. The biochemical tests performed at 6 weeks were compared between the groups to predict the presence of MBDP.

**Figure 1:**
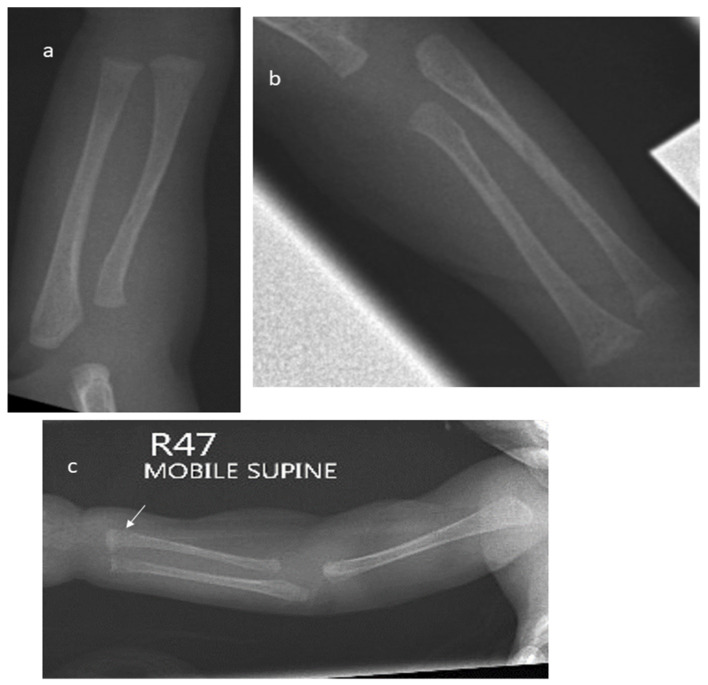
(**a**) X-ray of the right forearm in a 6-week-old infant born at 26 weeks showing changes of Grade 1 MBDP. (**b**) Wrist X-ray of an 8-week-old female infant born at 25 weeks showing cupping and fraying of metaphyses, Grade 2 MBDP. (**c**) X-ray of the right forearm in an 8-week-old infant born at 24 weeks showing a fracture at the distal end of the radius (marked by arrow).

The plain radiographs of all enrolled infants were reviewed by an independent paediatric radiologist who was blinded to the clinical details and biochemical test results. MBDP was identified and graded according to Koo et al. [19] as follows:

- No MBDP: normal density with white line at metaphyseal region
- Grade 1: loss of dense white line at metaphyses and thinning of cortex (Figure 1a)
- Grade 2: changes of grade 1 plus irregularity and fraying of metaphyses, with splaying and cupping (Figure 1b)
- Grade 3: bone changes seen in grade 1 and grade 2 in addition to fracture (Figure 1c)

### Statistics

Statistical analyses were conducted in Stata SE Version 14.2 and R Studio Version 4. Hypotheses were conducted at a significance level of 0.05 with a two-sided alternative. Summary statistics compared infants between the two groups. Normally distributed continuous variables were summarized by their mean and standard deviation, and differences assessed by a *t*-test. Non-normally distributed continuous variables were summarized by their median and interquartile range, and differences assessed by Wilcox’s rank sum test. Categorical variables were summarised by their proportions and counts, and differences assessed by Fisher’s exact test. Univariable and multivariable logistic regression models were used to assess the primary outcome, with Firth’s penalty applied to control for the low event rate in the second group. The multivariable model was adjusted for significant differences between the study cohorts. Odds ratios, 95% confidence intervals and *p*-values were reported. Missing data was low and not imputed. The biomarkers were assessed by a receiver operator characteristic (ROC) curve and sensitivity and specificity. 

## 3. Results

### 3.1. Participants

**Figure 2:**
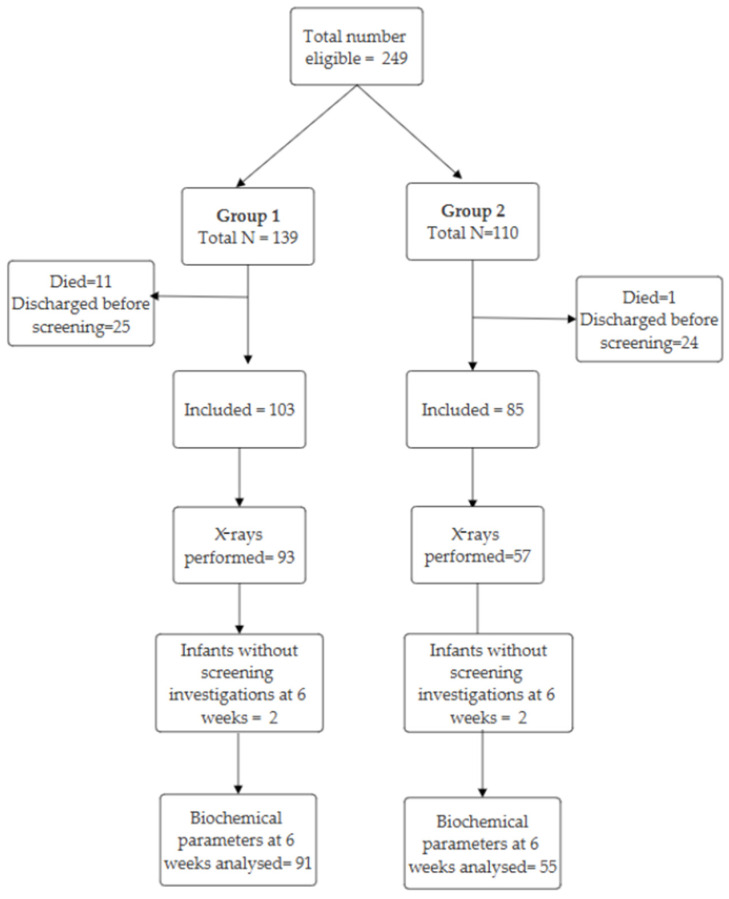
Study cohort in two epochs. Group 1 includes infants born in the first epoch, from 1 January 2016 to 31 December 2017, and Group 2 includes infants born in the second epoch, from 1 June 2018 to 31 May 2020.

Between 1 January 2016 and 31 May 2020, we identified 249 eligible preterm infants born <29 weeks’ gestation. Of these, 188 infants met the inclusion criteria (Figure 2). Ten infants in group 1 and seven infants in group 2 did not have blood and urine investigations to screen for MBDP at 6 weeks. The number of deaths in Epoch 1 in the study population was significantly higher than that in Epoch 2 (Epoch 1: 11/139, 7.9%; Epoch 2: 1/110, 0.9%, *p* < 0.01). We did not analyse the underlying causes of mortality in the two epochs as these infants were excluded from this study and this was not an objective of this study. There were no significant differences in the baseline characteristics of the infants that were included in the two groups except for a higher proportion of infants who received complete antenatal steroids and postnatal inhaled steroids in group 2 (Table 1).

### 3.2. Radiologic MBDP

In group 1, 90% (93/103) of infants had a plain radiograph of the wrist or the knee joint to look for MBDP compared to 67% (57/85) of infants in group 2. Table 2 shows the incidence and severity of MBDP between the two groups. No infants in group 2 had grade 2 or 3 MBDP. These differences are statistically significant (*p* < 0.01). Firth’s penalised logistic regression was used and adjustments were made for differences in completion of antenatal steroids and differences in the use of inhaled steroids. This result remained significant after adjusting for differences between the groups such as completion of antenatal steroids and use of inhaled steroids. When we consider any grade versus no MBDP, in the univariate model, the second epoch was assigned an odds ratio of 0.19 (0.09–0.40) with a *p*-value of <0.01; in the multivariate model, the second epoch was assigned an odds ratio of 0.39 (0.16–0.94) with a p-value of 0.04. When we consider Grade 2/3 versus No/Grade 1 MBDP, in the univariate model, the second epoch was assigned an odds ratio of 0.05 (0.01–0.37) with a *p*-value of <0.01; in the multivariate model, the second epoch was assigned an odds ratio of 0.10 (0.01–0.88) with a *p*-value of 0.03.

### 3.3. Biomarkers at MBDP Screening

Table 3 shows the differences in biomarkers between infants with and without MBDP for the whole cohort. Infants with grade 2 or 3 MBDP had lower Ca and P levels and higher levels of ALP. Although the difference in Ca levels achieved statistical significance, this difference is clinically not significant. PTH levels were slightly higher in grade 2 and 3 MBDP but this difference was statistically not significant. Vitamin D levels in both groups were low (<50 nmol/L).

Table 4 outlines the laboratory reference values for the tests conducted in this study.

### 3.4. Sensitivity and Specificity Analysis

**Figure 3:**
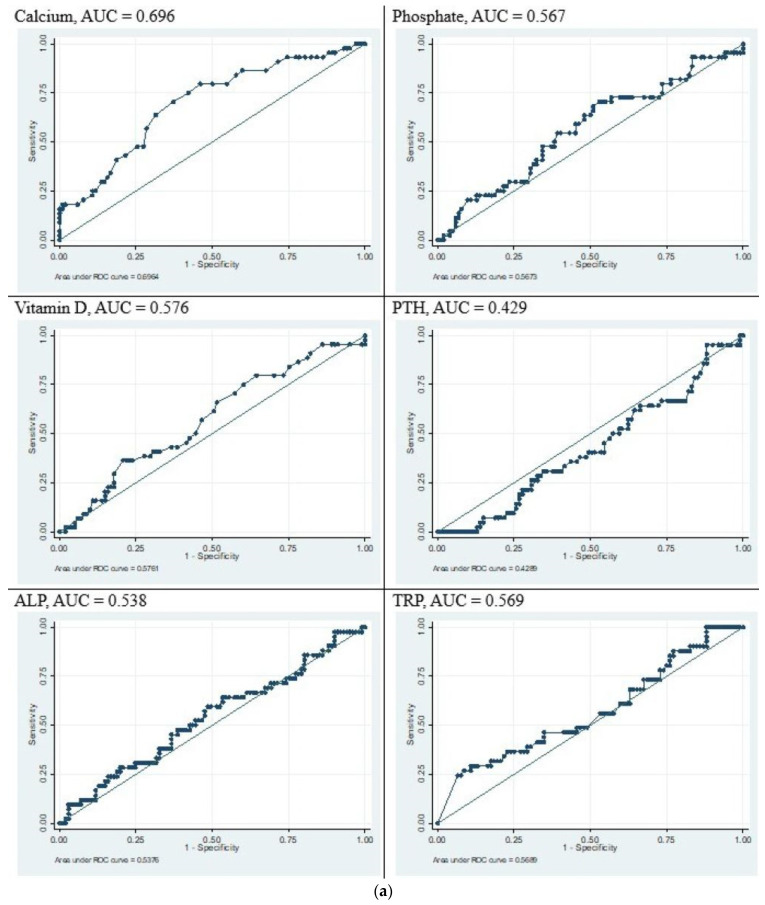

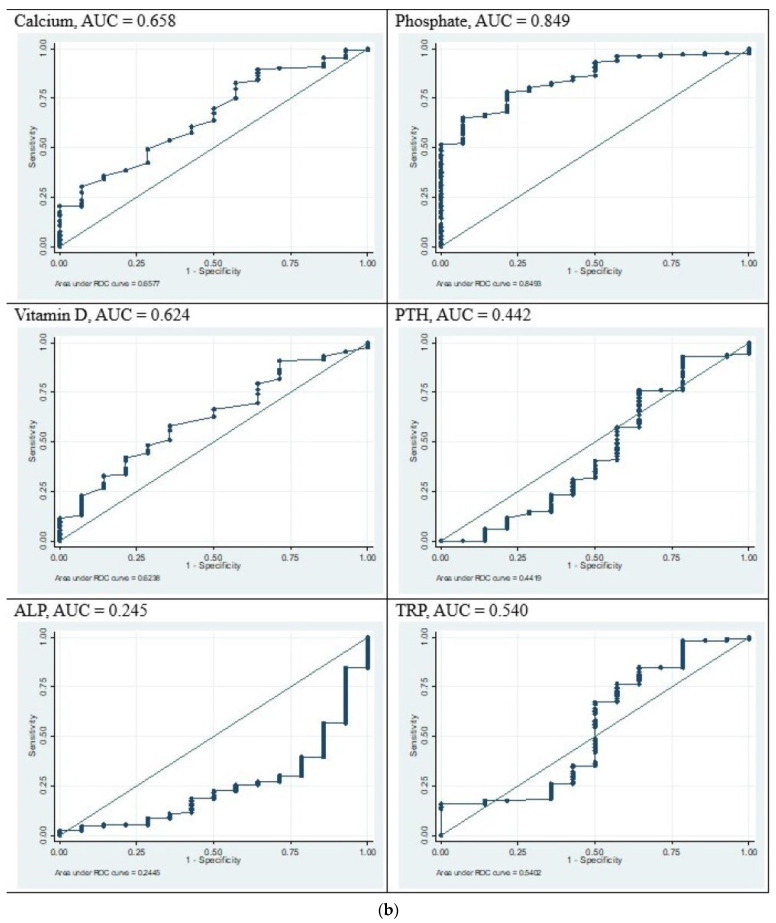
(**a**) ROC curves depicting correlation of biochemical tests with any grade of MBDP (grade 1/2/3). (**b**) ROC curves depicting correlation of biochemical tests with grade 2 or 3 MBDP.

All the biomarkers used to detect MBDP at 6 weeks of age were analysed with ROC curves (Figure 3a,b). Low serum phosphate was the best test characteristic for predicting grade 2 or 3 MBDP with an area under the curve of 0.849. None of the biochemical markers were shown to have satisfactory predictive value when predicting any grade of MBDP. Serum phosphate and ALP were both seen to have reasonably good predictive value for grade 2 and 3 MBDP. In our data, a phosphate value of <1.5 mmol/L had a specificity of 77% and sensitivity of 79%. A cut-off of >500 U/L for ALP had a specificity of 71% and sensitivity of 72%. A combination of the two parameters did not enhance the sensitivity or specificity.

### 3.5. Biochemical Parameters When Receiving PN Formulation

A comparison of serum Ca and P levels between the two groups, measured while the infants were receiving PN, is shown in Table 5. Both serum Ca and P levels were significantly higher in infants in group 2. Table 6 shows a comparison of the adverse events in the two groups. None of the infants in Group 2 showed hypocalcaemia. Although there was a significant increase in hypercalcaemia in the second group, this did not result in a significant increase in nephrocalcinosis at 6 weeks [22/95 (23%) in group 1 and 20/71(28%) in group 2, *p* = 0.99].

## 4. Discussion

This retrospective single-centre study found that PN formulation with higher calcium and phosphate concentrations and a change to organic phosphate reduced overall MBDP and eliminated severe (grade 2 and 3) MBDP. Overall serum Ca and P levels increased when using this PN formulation without any adverse effects such as hypophosphataemia [20] or nephrocalcinosis. In a retrospective study by Motokura et al. [21], lower rates of MBDP were reported in preterm infants receiving PN with higher P and Ca content, as seen in our study.

The improved antenatal steroid status in Group 2 may be attributed to improvements in antenatal management of pregnant women at risk for preterm birth. There was a notably significant reduction in mortality in Epoch 2 in comparison to Epoch 1. Although we did not analyse the causes of death in these infants, it is reasonable to speculate that the improved use of antenatal steroids that was noted in Epoch 2 may have had an impact on overall mortality [22].

An important change that occurred in our unit was the introduction of Pasteurized Donor Human Milk (PDHM) in 2018 which coincided with the second epoch. Despite the use of PDHM in group 2 as opposed to preterm formula in group 1, where the mother’s own milk was not available, the rates of MBDP in group 2 did not increase. Although donor human milk has a lower mineral content compared to preterm formula, long-term follow-up studies of infants randomized to human milk versus formula milk have shown improved bone mineral content in adulthood in subjects who received predominantly human milk. This phenomenon has been attributed to the presence of non-nutritive factors in breast milk [23]. The duration of parenteral nutrition and the days to fortification of feeds after birth, which have historically been known to be important risk factors for MBDP, were not different between the two epochs. It is possible that use of PDHM and PN with increased mineral content may have contributed to the reduction in rates of severe MBDP in our cohort.

Amongst the various tests conducted to detect MBDP, we found that low serum phosphate levels of <1.5 mmol/L had the best specificity (77%) and sensitivity (79%) for detecting grade 2 and 3 MBDP. ALP levels were higher in the group that had grade 2 to 3 MBDP. ALP > 500 U/L showed a specificity of 71% and sensitivity of 72%. These findings are similar to those of other studies where ALP > 500 and *p* < 1.44 mmol/L were found to be useful in predicting MBDP [24,25].

Although ALP is commonly used as a marker of MBDP, evidence for its utility in diagnosing MBDP is conflicting. Backstrom et al. [26] correlated bone densitometry using DEXA at 3 months with serum ALP and phosphate levels in preterm infants. A combination of total ALP > 900 IU/L and serum inorganic phosphate concentrations <1.8 mmol/L yielded a sensitivity of 100% at a specificity of 70%. Early screening with ALP has been proposed by several authors with a view to preventing MBDP [25,27]. On the contrary, Faerk et al. [28] showed that bone mineral content as determined by DEXA did not correlate with serum ALP or serum phosphate at term-equivalent age.

Our cohort of infants had an overall low baseline level of vitamin D, despite the routine supplementation of all neonates with 400 IU vitamin D per day based on the existing guidelines. Vitamin D levels did not correlate significantly with diagnosing MBDP, which is consistent with findings from others that vitamin D levels are usually normal in infants with MBDP [29]. PTH plays an important role in the pathophysiology and diagnosis of MBDP. It helps to distinguish between calcipenic and phosphopenic states contributing to MBDP, thereby guiding management of MBDP. Elevated levels of PTH suggest a calcipenic state. Conversely, where phosphate deficiency exists, PTH levels may be normal. A retrospective study of serum PTH levels in infants with radiological evidence of MBDP showed a high incidence of hyperparathyroidism (82%) in infants with radiological MBDP [30]. In our study, PTH levels were not seen to correlate with radiological MBDP. We speculate that this may be due to the presence of both calcipenia and phosphopenia in the etiopathogenesis of MBDP in our cohort.

Tubular reabsorption of phosphate was not found to be a significant predictor of MBDP in our study. Urinary calcium and phosphate excretion may be used as a surrogate for PTH in determining the presence of secondary hyperparathyroidism. A high TRP with low phosphate indicates a phosphopenic state, while a low or normal TRP with low phosphate may indicate hyperparathyroidism secondary to calcium deficiency. It is important to note that extremely preterm infants can have immature kidneys that may result in increased urinary excretion of phosphate despite a relative phosphate deficiency state. We did not measure urinary calcium as part of MBDP workup in our cohorts.

### 4.1. Limitations

This study shares the limitations of retrospective studies in the lack of control over the study population with unequal data sets and its implications on the comparisons between the two groups. Many infants in group 2 did not have routine screening X-rays. This may reflect the variation in practice within our unit due to a lack of consensus on screening guidelines and the definition of MBDP. We also acknowledge the limitations of X-rays in diagnosing early MBDP before significant demineralization has occurred. In our study, we considered X-rays as the gold standard for diagnosis of MBDP; hence we may have missed some infants with MBDP.

### 4.2. Strengths

This is the first study looking at the impact of two PN formulations on bone health in a large cohort of preterm infants. An important strength in the methodology was the independent reporting of X-rays by a paediatric radiologist who was blinded to the clinical details of the patients. The results of this study may be generalizable to neonatal intensive care units with similar nutritional practices to ours such as early parenteral nutrition, routine fortification of enteral feeds, and preferential administration of mother’s milk as opposed to formula.

### 4.3. Future Directions

The composition of PN solutions has undergone further revision with a revised Ca:P ratio in 2022. In addition, we have revised our enteral nutrition guidelines, implementing slightly earlier fortification of feeds and increasing the dose of supplemental vitamin D. We are also revising our bone health screening protocol using serum phosphate and ALP levels as first-tier screening investigations, with further testing, including X-rays, reserved for infants showing abnormal results in these tests as recommended by other authors [11,12]. We plan to evaluate the effects of these interventions in a future audit.

## 5. Conclusions

We have demonstrated an overall reduction in MBDP and elimination of more severe grades of MBDP with the use of a PN formulation with higher mineral content. We believe that earlier fortification of feeds and an increased dose of vitamin D may further improve bone health in extremely preterm infants. Serum phosphate and ALP were the best predictors of radiological MBDP.

## Figures and Tables

**Table 1:** Baseline characteristics of infants in two PN epochs.

Characteristic	Group 1 (*n* = 103)	Group 2 (*n* = 85)	*p*
Gestational age (weeks)	26 (25–27.5) ^†^	26 (25–27) ^†^	0.90
Birthweight (g)	898 (755–1040) ^†^	940 (763–1040) ^†^	0.36
Birthweight (percentile)	60 (33.5–77) ^†^	63 (39–80) ^†^	0.48
Ethnicity			
White	59 (57%)	41 (48%)	0.24
Asian	29 (28%)	34 (40%)	
Other	15 (15%)	10 (12%)	
Abnormal Dopplers	11 (15%)	9 (12%)	0.81
Antenatal steroids received			
None	7 (7%)	6 (7%)	
Incomplete	42 (41%)	24 (28%)	
Complete	29 (28%)	42 (49%)	0.02
More than 7 days	25 (24%)	13 (15%)	
Multiple gestation			
Singleton	73 (71%)	58 (68%)	0.21
Twins	20 (29%)	24 (28%)	
Triplets	0 (0%)	3 (4%)	
Maternal pregnancy induced hypertension (PIH)	26 (25%)	17 (20%)	0.49
Chronic lung disease	56 (54%)	41 (48%)	0.49
Necrotising enterocolitis (NEC)	11 (11%)	7 (8%)	0.92
Patent ductus arteriosus	93 (90%)	76 (89%)	1.00
Duration of parenteral nutrition (days)	23.7 (15.4–34.7) ^†^	21.1 (15.8–28.4) ^†^	0.32
Postnatal systemic steroids	26 (25%)	30 (35%)	
Inhaled steroids	6 (6%)	18 (21%)	0.01
Duration of loop diuretic therapy (days)	1 (0–4) ^†^	2 (0–5) ^†^	0.23
Placenta histology			
Normal	12 (13%)	21 (23%)	0.07
Chorioamnionitis	60 (65%)	46 (58%)	
Ischemia	20 (22%)	12 (15%)	
Sex			
Male	46 (45%)	45 (53%)	0.31
Days to full fortification *	25 (18–35) ^†^	24 (19–31) ^†^	0.48

^†^ Values depicted as median and interquartile range. * Full fortification defined as fortification to 24 calories per oz (29.6 mL) of breast milk.

**Table 2:** MBDP severity between the two groups.

MBDP Severity	Group 1 (*n* = 93)	Group 2 (*n* = 57)	Total Cohort
No MBDP	16 (17%)	30 (53%)	46 (31%)
Grade 1	63 (68%)	27 (47%)	90 (60%)
Grade 2	13 (14%)	0 (0%)	13 (9%)
Grade 3	1 (1%)	0 (0%)	1 (0.67%)

**Table 3:** Comparison of serum and urine markers between infants with and without MBDP.

Test	Number of Infants with Data Available	No MBDP (*n* = 44) †	Grade 1 MBDP (*n* = 88) †	Grade 2/3 MBDP (*n* = 14) †	*p*
Calcium (mmol/L)	146/146	2.68 (2.64–2.78)	2.62 (2.56–2.70)	2.60 (2.47–2.66)	<0.01
Phosphate (mmol/L)	146/146	1.83 (1.46–2.04)	1.80 (1.53–2.02)	1.34 (1.18–1.47)	<0.01
Vitamin D (nmol/L)	145/146	46 (40–62)	44 (37–56)	41 (31–48)	0.16
PTH (pmol/L)	140/146	7.15 (3.9– 11.4)	8.00 (5.50–13.95)	9.45 (4.60–17.30)	0.37
ALP (U/L)	146/146	426 (332–567)	384 (329–506)	553 (484–817)	<0.01
TRP (%)	122/146	95 (91–100)	95 (91–98)	95 (88–100)	0.44

† Values depicted as median and interquartile range.

**Table 4:** Reference values for the investigations performed for screening of MBDP.

Test	Reference Values
Serum corrected Calcium	1.95–2.8 mmol/L
Serum phosphate	1.45–2.5 mmol/L
Alkaline Phosphatase (ALP)	120–650 U/L
Parathyroid Hormone (PTH)	1.6–7.5 pmol/L
25-Hydroxy(OH) Vitamin D	>50 nmol/L
Tubular reabsorption of phosphate (TRP)	>95%
Radiograph of the wrist/knee joint	Radiologist review for MBDP
Renal ultrasound for nephrocalcinosis	Radiologist report

**Table 5:** Comparison of serum calcium and phosphate levels during the administration of PN.

	Group 1 (*n* = 103)	Group 2 (*n* = 85)	*p*
Calcium			
Low	9 (9%)	0 (0%)	<0.01
Normal	74 (72%)	53 (62%)	
High	20 (19%)	32 (38%)	
Phosphate			
Low	53 (51%)	21 (25%)	<0.01
Normal	44 (43%)	60 (71%)	
High	6 (6%)	4 (5%)	

**Table 6:** Comparison of adverse events between groups with change in PN.

	Group 1 (*n* = 103)	Group 2 (*n* = 85)	*p*
Hypocalcaemia ^#^	9 (9%)	0 (0%)	<0.01
Hypercalcaemia ^#^	20 (19%)	32 (38%)	<0.01
Hypophosphataemia ^#^	53 (51%)	21 (25%)	<0.01
Nephrocalcinosis *	22/95 (23%)	20/71 (28%)	0.99

^#^ Blood investigations studied in the first 2 weeks of life. * Renal Ultrasound for nephrocalcinosis at 6 weeks.

## Data Availability

All data generated or analysed during this study are included in this published article.
